# Genome-Wide Identification and Functional Characterization of the *TIFY* Gene Family Associated with Abiotic Stress and Hormone Response in *Carthamus tinctorius* L.

**DOI:** 10.3390/ijms27198752

**Published:** 2026-09-30

**Authors:** Lina Wang, Lei Li, Xiaoyu Su, Yiwen Cao, Dandan Lu, Yaling Yang, Yao Sun, Mengfan Su, Chunming Li, Yongliang Yu, Zhengwei Tan, Huizhen Liang

**Affiliations:** 1 Institute of Chinese Herbal Medicines, Henan Academy of Agricultural Sciences, Zhengzhou 450002, China; hnndlina@163.com (L.W.); 15136189572@163.com (L.L.); suxiaoyu@hnagri.org.cn (X.S.); yiwencao96@163.com (Y.C.); sufancy1669@163.com (M.S.); lchm1212@hnagri.org.cn (C.L.); yyl790721@126.com (Y.Y.); 2 Provincial Key Laboratory of Conservation and Utilization of Traditional Chinese Medicine Resources, Institute of Chinese Herbal Medicines, Henan Academy of Agricultural Sciences, Zhengzhou 450002, China

**Keywords:** *Carthamus tinctorius* L., *CtTIFY* gene family, abiotic stress, hormone response bioinformatic analysis, transcript expression levels

## Abstract

Jasmonate-ZIM domain (JAZ) proteins act as core negative regulators of jasmonic acid signaling and mediate plant stress adaptation, developmental progression, and secondary metabolism. Safflower (*Carthamus tinctorius* L.) is an economically important Asteraceae crop with medicinal, edible oil, and ornamental values; however, the evolutionary characteristics and molecular functions of its *TIFY* gene family remain poorly characterized. In the present study, we performed a genome-wide identification of the safflower *CtTIFY* family, followed by systematic analyses of phylogenetic relationships, chromosomal distribution, gene synteny, structural features, conserved motifs, and promoter cis-regulatory elements. Transcriptome profiling, quantitative real-time PCR (qRT-PCR) validation under multiple abiotic stresses and methyl jasmonate treatment, and subcellular localization assays were further conducted to explore divergence in transcript expression levels among family members. A total of 14 *CtTIFY* genes were identified and categorized into three subfamilies: JAZ, TIFY, and ZML. Segmental duplication was the primary driving force for *CtTIFY* family expansion, and distinct structural and physicochemical divergences were detected among different subfamilies. *CtTIFY* genes harbor abundant cis-elements responsive to stresses, hormones, and light signals, and exhibit transcriptional correlations with safflower floral development, seed germination, and seed-oil accumulation. Among them, *CtJAZ1*, *CtJAZ6*, and *CtJAZ7* represent key candidate genes showing transcriptional responses to JA signals and multiple abiotic stresses. Subcellular localization showed that *CtTIFY* proteins are predominantly nuclear-localized with potential nucleocytoplasmic shuttling. This study systematically characterizes the evolutionary dynamics and divergence in expression levels of the safflower *CtTIFY* family, identifies candidate genes associated with stress response and quality-related processes, and provides fundamental genetic resources for future safflower molecular-breeding research.

## 1. Introduction

Safflower (*Carthamus tinctorius* L.) is an annual medicinal and oil crop belonging to the Asteraceae family, with a cultivation history of over 4000 years in China. Its florets are rich in flavonoids and phenolic compounds, and its seeds accumulate high levels of linoleic acid, conferring considerable application values in pharmaceutical production, food processing, edible oil extraction, and ornamental horticulture [1,2,3,4]. Safflower is mainly cultivated in arid and semi-arid regions, and long-term natural domestication has endowed this crop with strong tolerance to drought, salinity, and low temperature [5,6]. Therefore, systematically dissecting the molecular basis of safflower stress responses and exploring key genes associated with stress tolerance and quality traits are essential for breeding high-quality and stress-resilient safflower varieties.

Jasmonic acid (JA) and its derivative methyl jasmonate (MeJA) are essential endogenous plant hormones that broadly modulate plant adaptive responses to diverse abiotic stresses, including cold, drought, and salinity. In addition, the JA signaling pathway participates in the regulation of plant growth, organ morphogenesis, and the biosynthesis of flavonoid and phenolic secondary metabolites, serving as a core molecular hub linking environmental adaptation and developmental regulation [7,8,9,10]. JAZ proteins are the primary negative regulators of JA signaling and possess conserved structural domains, including an N-terminal TIFY/ZIM motif and a C-terminal Jas domain [11]. The N-terminal TIFY/ZIM motif mediates homo- and heterodimerization of JAZ proteins, whereas the C-terminal Jas domain specifically interacts with the JA receptor CORONATINE INSENSITIVE 1 (COI1), thereby triggering ubiquitin-26S proteasome-mediated degradation of JAZ repressors [12]. This degradation process relieves the inhibitory effects on downstream transcription factors (e.g., MYC and EIL) and subsequently activates JA signaling cascades [11,13]. Accumulating evidence has demonstrated that JAZ proteins interact with key components of multiple hormone signaling pathways, including abscisic acid (ABA) and ethylene, forming a complex hormone crosstalk network to coordinately regulate plant stress tolerance and growth plasticity [12,14,15,16].

Genome-wide identification and characterization of *TIFY* gene families have been extensively reported in multiple plant species, including *Arabidopsis thaliana* [17], rice [18], and sunflower [19], revealing substantial sequence and functional divergence among JAZ paralogs across different species. In Arabidopsis, *AtJAZ* genes maintain plant growth and reproductive stability by alleviating immune-induced metabolic disorders [20]. In wheat, *TaJAZ1* modulates the expression of ABA-responsive genes and negatively regulates ABA-mediated inhibition of seed germination [21]. In rice, *OsJAZ1* acts as a vital transcriptional regulator in JA signaling and enhances drought tolerance through interaction with *OsbHLH148*, while the *OsJAZ1*–*OsCOI1b* module mediates JA-dependent root and floral development [22]. Soybean *GsJAZ2* significantly improves plant tolerance to salt and alkali stresses [23]. Furthermore, *PnJAZ1* from *Pohlia nutans* mediates synergistic crosstalk between JA and ABA signaling to promote plant growth under salt stress [24]. Collectively, *TIFY* genes function as critical molecular links bridging plant growth metabolism and stress adaptation [25].

The plant TIFY gene family comprises three subfamilies: JAZ, TIFY, and ZML. Notably, TIFY and ZML subfamily members lack the canonical Jas domain that characterizes typical JAZ proteins, indicating structural diversification within the TIFY superfamily.

Despite extensive studies on plant *TIFY* families, systematic genomic and transcriptional analyses of the safflower *CtTIFY* family remain limited. The gene number, evolutionary features, structural differentiation, spatiotemporal and stress-responsive expression patterns of safflower *CtTIFY* genes remain poorly understood, which restricts our comprehensive understanding of JA-associated stress responses and quality-related regulatory mechanisms in safflower. To address this research gap, the present study performed a comprehensive genome-wide analysis of the *CtTIFY* family, including phylogenetic evolution, chromosomal localization, gene synteny, structural features, conserved motifs, and promoter cis-elements. Combined with transcriptome profiling and qRT-PCR validation under cold, salt, PEG-6000, and MeJA treatments, as well as subcellular localization assays, we systematically characterized the evolutionary features and transcript expression-level profiles of *CtTIFY* members. This study provides a theoretical reference and valuable gene resources for molecular breeding of stress resistance and quality improvement in safflower.

## 2. Results

### 2.1. Genome-Wide Identification and Physicochemical Characterization of CtTIFY Genes

A total of 14 *CtTIFY* family members were identified from the safflower genome, including *CtZML1*, *CtZML2*, *CtZIM*, *CtTIFY1*, *CtTIFY2*, and *CtJAZ1*–*CtJAZ9*. Significant subfamily-specific divergences in physicochemical properties were observed (Table 1). The amino acid length of CtTIFY proteins ranged from 121 to 412 aa, with MW varying from 15.0 to 41.9 kDa. CtJAZ2 had the shortest sequence, CtTIFY1 had the longest sequence, and CtZML2 exhibited the largest MW. CDS length was positively correlated with amino acid number, with short-peptide CDSs < 400 bp and long-protein CDSs > 1000 bp.

The theoretical isoelectric point (pI) values ranged from 5.36 to 9.75. Five members (CtZML1, CtZML2, CtZIM, CtJAZ5, CtJAZ6) encoded acidic proteins, among which CtJAZ6 had the lowest pI. The remaining nine proteins were alkaline, and CtJAZ2 showed the highest alkalinity. All CtTIFY proteins had negative GRAVY values, confirming their hydrophilic nature. CtJAZ4 and CtJAZ7 exhibited the highest hydrophilicity, while CtJAZ1 and CtJAZ2 showed the lowest.

Protein stability analysis showed that only CtZIM and CtJAZ5 had instability indices below 40, indicating stable characteristics in vitro, whereas the other 12 members were unstable, with CtJAZ1 having the highest instability index. The aliphatic index ranged from 53.26 to 78.26, suggesting variable thermal stability; CtJAZ8 had the highest thermal stability, and CtZIM had the lowest.

### 2.2. Phylogenetic Analysis of the CtTIFY Family

To clarify the phylogenetic classification and evolutionary relationships of the *CtTIFY* family, an NJ tree was constructed using TIFY proteins from safflower, Arabidopsis, rice, and sunflower. All TIFY homologs were divided into eight clades corresponding to three subfamilies: JAZ, TIFY, and ZML (Figure 1). The 14 *CtTIFY* members were unevenly distributed across eight clades, with no safflower homologs in Clade 4, indicating species-specific evolutionary divergence.

The canonical JAZ subfamily displayed clear clustering patterns: *CtJAZ6*, *CtJAZ8*, and *CtJAZ9* belonged to Clade 1; *CtJAZ3* was uniquely clustered in Clade 2; *CtJAZ4*, *CtJAZ5*, and *CtJAZ7* were grouped into Clade 3; *CtJAZ1* and *CtJAZ2* were distributed in Clade 5. For the TIFY subfamily, *CtTIFY1* and *CtTIFY2* clustered specifically in Clade 6. ZML subfamily genes (*CtZIM*, *CtZML1*, *CtZML2*) were concentrated in Clade 7 and Clade 8 (Figure 1).

Each clade contained homologs from both monocot and dicot species, indicating that the functional differentiation of TIFY subfamilies occurred before the monocot–dicot divergence, and that the core evolutionary characteristics of this family are relatively conserved across plant lineages. In addition, the three safflower subfamilies (JAZ, TIFY, and ZML) exhibited distinct clustering boundaries and stable topological structures in the phylogenetic tree, supporting the reliability of the subfamily classification performed in the present study.

### 2.3. Chromosomal Localization and Collinearity Analysis

The 14 *CtTIFY* genes were unevenly distributed on 12 safflower chromosomes. Only chromosome CtAH01 harbored a tandem gene cluster (*CtJAZ1*/*CtJAZ2*), while other chromosomes contained single *CtTIFY* loci (Figure 2a). Intraspecific collinearity analysis identified six segmental duplication gene pairs: *CtJAZ4*/*CtJAZ5*, *CtJAZ4*/*CtJAZ7*, *CtJAZ5*/*CtJAZ7*, *CtJAZ6*/*CtJAZ8*, *CtJAZ8*/*CtJAZ9*, and *CtZML1*/*CtZML2* (Table S1). No effective tandem duplication events were detected, indicating that segmental duplication is the dominant driving force for *CtTIFY* family expansion (Figure 2b).

Interspecific collinearity analysis showed that safflower shared the most orthologous pairs with sunflower, followed by Arabidopsis, and the fewest with rice, consistent with their phylogenetic relationships. Multiple one-to-many homologous relationships further confirmed the close evolutionary affinity between safflower and Asteraceae species (Figure 2c). All homologous gene pairs had *Ka/Ks* < 1 (Table S1), demonstrating that the *CtTIFY* family has undergone strong purifying selection during evolution, with core functional domains highly conserved.

### 2.4. Gene Structure and Conserved Motif Analysis

Genes within the same phylogenetic clade exhibited highly conserved exon–intron arrangements, whereas significant structural divergence existed among different subfamilies (Figure 3a,b). ZML and TIFY subfamily genes had longer sequences and more complex exon–intron structures, while canonical *JAZ* genes had compact structures with fewer exons. *CtZML2* had the longest full-length sequence with an extended 3′ UTR, and variable UTR distributions among members contribute to transcriptional regulatory diversity.

A total of 10 conserved motifs were identified in CtTIFY proteins (Figure 3c,d). Motif 1 was universally conserved in all members, serving as the core functional domain for protein interaction and JA signal transduction. Subfamily-specific motif compositions were characterized: canonical JAZ members contained Motif 1/2/7 and lacked Motif 5/6/9/10; ZML subfamily specifically harbored Motif 3/4/9; TIFY subfamily was enriched in Motif 3/6. Minor motif deletion variations (e.g., Motif 2 loss in *CtJAZ5*) provide a structural basis for the functional differentiation of *CtJAZ* members.

### 2.5. Analysis of Cis-Acting Elements in CtTIFY Promoters

In total, 400 cis-elements belonging to 35 categories were identified in *CtTIFY* promoter regions, which were classified into light-/development-, abiotic stress-, and hormone-responsive modules (Figure 4). Light-responsive elements were the most abundant, with G-box and GT1-motif as core elements. *CtJAZ5* contained 16 G-box copies and *CtJAZ1* contained six GT1-motif copies, implying the involvement of *CtTIFY* genes in light-mediated growth and secondary metabolism.

Six types of stress-responsive elements were detected. The anaerobic induction element ARE was the most abundant (10.25% of total elements). All *CtTIFY* genes except *CtJAZ8* harbored ARE elements, with *CtJAZ1* and *CtJAZ6* having the highest copy numbers. The drought-responsive MBS motif was widely distributed across the family (Figure 4), indicating universal stress-responsive potential.

Nine types of hormone-responsive elements were identified. MeJA-responsive TGACG-motif/CGTCA-motif existed in all members except *CtTIFY2*. ABA-responsive ABRE elements accounted for 20% of total elements and were present in all genes except *CtJAZ4*. *CtJAZ3*, *CtJAZ5*, and *CtJAZ6* had significantly more ABRE copies than other members. Overall, *CtJAZ5* and *CtJAZ6* had the most abundant regulatory elements, while *CtTIFY2* had the fewest (Figure 4), indicating distinct signal integration capacities and functional differentiation among family members.

### 2.6. Spatiotemporal Expression Patterns of CtTIFY Genes Based on Transcriptome Data

Transcriptome analysis revealed distinct spatiotemporal expression patterns of *CtTIFY* genes across different flower colors, seed developmental stages, oil accumulation processes, and floral developmental phases (Figure 5). Four flower color varieties exhibited specific expression profiles, *CtJAZ1*/*CtJAZ5*/*CtJAZ6*/*CtJAZ7* were highly expressed in W; *CtZIM* and *CtTIFY1* were dominant in Y; *CtJAZ1*/*CtJAZ3*/*CtJAZ9* exhibited high expression levels in LR; *CtJAZ2*/*CtJAZ8*/*CtZML2* showed the highest expression in DR (Figure 5a).

Seed germination analysis revealed stage-specific expression patterns: *CtZML1* was specifically induced at 1 DAG; *CtTIFY1* peaked at 3 DAG; *CtTIFY2* was highly expressed at 5 and 7 DAG; *CtZML2* and *CtJAZ6* were significantly upregulated at 10 DAG, pointing to potential transcriptional-level associations of ZML and TIFY subfamily members with seed germination and seedling establishment. For seed oil traits, *CtJAZ1* and *CtJAZ2* showed high expression in LL_DAF10 compared with HL_DAF10, suggesting a possible transcriptional link between JA-related genes and the regulation of fatty acid biosynthesis. During flower development, *CtTIFY2*, *CtJAZ4*, and *CtJAZ3* were predominantly expressed in SBS, MBS, and DFS, respectively (Figure 5b), revealing stage-specific transcriptional patterns of *CtTIFY* members during floral development.

### 2.7. Expression Profiles of CtTIFY Genes Under Abiotic Stresses

qRT-PCR analysis revealed distinct stress response patterns of *CtTIFY* genes under cold, salt, and PEG-6000 treatments (Figure 6). Under cold stress, *CtTIFY* members fell into two response modes: six genes (*CtJAZ1*, *CtJAZ7*, *CtJAZ8*, *CtTIFY1*, *CtZML1*, *CtZML2*) were rapidly induced and peaked at 3–6 h, showing early-stage cold-responsive transcriptional patterns; seven genes were continuously down-regulated, while only *CtJAZ2* was significantly up-regulated at 48 h (Figure 6a).

Under salt stress, most *CtTIFY* genes showed transient high expression at 1 h. *CtJAZ1*, *CtJAZ4*, *CtTIFY1*, *CtTIFY2*, *CtZIM*, and *CtZML2* exhibited the highest induction folds, displaying early-stage salt-inducible transcriptional characteristics. In contrast, *CtJAZ3*, *CtJAZ6*, and *CtJAZ9* were continuously suppressed throughout treatment (Figure 6b).

Under PEG-6000-induced osmotic stress, nine *CtTIFY* genes (*CtJAZ1*, *CtJAZ2*, *CtJAZ3*, *CtJAZ4*, *CtJAZ7*, *CtJAZ8*, *CtZIM*, *CtZML1*, *CtZML2*) were rapidly up-regulated within 1–3 h, exhibiting early-stage osmotic-stress-responsive transcription, whereas four genes were continuously inhibited. Collectively, *CtJAZ1*, *CtJAZ7*, *CtZML1*, and *CtZML2* exhibited broad-spectrum transcriptional responses to multiple abiotic stresses (Figure 6c).

### 2.8. Expression Patterns of CtTIFY Genes in Corollas Under MeJA Treatment

The abundant MeJA-responsive cis-elements in *CtTIFY* promoters suggest potential JA-related transcriptional responsiveness in floral tissues. qRT-PCR results confirmed significant temporal response differentiation among 14 *CtTIFY* members under exogenous MeJA treatment (Figure 7 and Figure S1). *CtJAZ1*, *CtJAZ6*, and *CtJAZ8* were continuously and significantly induced by MeJA, showing sustained strong transcriptional induction by MeJA in corollas. In contrast, *CtJAZ4*, *CtJAZ7*, *CtTIFY1*, and *CtZIM* were markedly repressed. *CtJAZ5* maintained stable expression, while *CtJAZ9* showed delayed upregulation at 6–12 h (Figure 7). These results reveal divergent transcriptional responses of *CtTIFY* members to MeJA treatment in floral organs.

### 2.9. Subcellular Localization of CtTIFY Proteins

Subcellular localization assays showed that the 35S::GFP control displayed ubiquitous fluorescence in the nucleus and cytoplasm of *Nicotiana benthamiana* epidermal cells. In contrast, all CtTIFY-GFP fusion proteins exhibited predominant nuclear localization, with weak cytoplasmic signals observed for partial members (Figure 8). These results confirm that CtTIFY proteins are primarily nuclear-localized, consistent with the subcellular features of transcriptional regulators, and may undergo dynamic nucleocytoplasmic shuttling for signal regulation.

## 3. Discussion

JAZ proteins are core negative regulators of plant JA signaling and play conserved roles in modulating stress adaptation, hormone crosstalk, and secondary metabolic reprogramming. The evolutionary and functional characteristics of JAZ families have been well documented in multiple plant species, including Arabidopsis [17], rice [18], wheat [26], soybean [27], Chinese cabbage [28], and sunflower [19]. However, systematic research on the safflower *CtTIFY* family has been lacking. In this study, we comprehensively identified 14 *CtTIFY* members and systematically analyzed their evolution, structure, characteristics of transcript expression levels, spatiotemporal expression, stress and hormone responses, and subcellular localization. This study complements the research system of Asteraceae *TIFY* families and provides new insights into JA-mediated regulatory expression patterns in safflower.

Physicochemical analysis (Table 1) showed that only *CtZIM* and *CtJAZ5* were stable proteins in vitro, consistent with previous reports in Arabidopsis [17], Phalaenopsis [29], and cotton [30]. As transient repressors of JA signaling, unstable TIFY proteins can be rapidly degraded upon stress or hormonal stimulation to activate JA cascades, while stable members maintain growth homeostasis under normal conditions [25,31]. The coexistence of stable and unstable *CtTIFY* proteins enables the family to coordinate developmental homeostasis and rapid stress responses, forming a functionally complementary regulatory module.

Phylogenetic (Figure 1) and collinearity (Figure 2) analyses classified the *CtTIFY* family into three conserved subfamilies with no safflower-specific clades. All homologous gene pairs had *Ka/Ks* < 1 (Table S1), indicating strong purifying selection and conserved core domains, which is consistent with the sunflower *HaJAZ* family [19]. Segmental duplication rather than tandem duplication dominates *CtTIFY* family expansion, which facilitates functional refinement and differentiation while maintaining structural integrity, implying adaptive evolutionary characteristics of safflower *TIFY* genes during Asteraceae diversification.

Gene structure and motif (Figure 3) analyses confirmed high structural conservation within subfamilies and significant divergence among subfamilies, consistent with previous studies [18,19]. Motif 1 is a universal core domain essential for JA signal transduction and protein interaction (Figure 3). Subfamily-specific motif variations provide structural basis for the functional divergence of *CtTIFY* genes in development, metabolism, and stress response.

Promoter cis-element (Figure 4) analysis indicated that *CtTIFY* genes integrate light, stress, and hormone signals, serving as multi-signal regulatory hubs. The enrichment of ABRE elements in *CtJAZ3*, *CtJAZ5*, and *CtJAZ6* supports their potential roles in JA-ABA crosstalk-mediated stress tolerance [15,32,33]. Widespread light-responsive elements further link JAZ signaling to light-regulated flower color formation and seed oil accumulation, revealing dual functions in stress-resistance and quality regulation.

Spatiotemporal transcriptome profiling (Figure 5) revealed transcriptional-level associations of *CtTIFY* genes with safflower flower development, seed germination, and oil accumulation, which is consistent with previously reported TIFY functions in plant development and lipid metabolism [10,12,25,34]. Transcription-based evidence points to potential subfamily-level differences in expression patterns: ZML and TIFY subfamily members show preferential expression during seed germination and seedling development, whereas canonical JAZ members exhibit high transcript abundance in floral organs and for secondary-metabolism-related processes. Differential expression across flower-color varieties offers novel clues for dissecting the regulatory mechanisms underlying safflower flower color.

qRT-PCR assays (Figure 6) showed that *CtJAZ1*, *CtJAZ7*, *CtZML1*, and *CtZML2* display broad-spectrum transcriptional responses to multiple abiotic stresses, with conserved expression patterns under stress treatments. Stress-repressed *CtTIFY* genes may reflect a potential trade-off between growth and defense at the transcript level, which resembles well-known adaptive strategies described in other plant species [32]. MeJA treatment (Figure 7) further identified *CtJAZ1*, *CtJAZ6*, and *CtJAZ8* as prominent JA-responsive candidates in floral tissues, providing candidate genes for future investigation of JA-mediated flower-quality regulation.

Subcellular localization (Figure 8) further identified *CtJAZ1*, *CtJAZ6*, and *CtJAZ8* as key JA-responsive genes in floral tissues, confirmed that CtTIFY proteins are predominantly nuclear-localized with weak cytoplasmic signals, similar to JAZ proteins from *Arabidopsis thaliana* and sunflower [19,20], indicating dynamic nucleocytoplasmic shuttling, which refines the JA signaling regulatory model in Asteraceae.

Overall, the safflower *CtTIFY* family exhibits both evolutionary conservation and divergence in expression levels, with diverse transcript profiles linked to stress adaptation, developmental processes, and quality-related traits. This study provides a reliable theoretical reference and valuable gene resources for future safflower molecular-breeding efforts.

## 4. Materials and Methods

### 4.1. Plant Materials and Stress Treatments

The safflower cultivar “Yuhonghua No. 1”, bred by the Institute of Chinese Herbal Medicines, Henan Academy of Agricultural Sciences, was used as the experimental material. This variety exhibits wide adaptability with documented drought- and salt-tolerant agronomic performance, and is used for both flower- and seed-related studies. Uniform and healthy safflower seeds were surface-sterilized and germinated in vermiculite substrate. Seedlings at six days after emergence were hydroponically cultured in half-strength Hoagland’s solution in an artificial climate chamber (Henan Academy of Agricultural Science, Zhengzhou, China) under controlled conditions: 25 °C day/20 °C night, 16 h light/8 h dark photoperiod, light intensity of 200 μmol·m^−2^·s^−1^, and 60–70% relative humidity. Three-leaf-stage seedlings with consistent growth status were selected for abiotic stress treatments, with normally cultured seedlings set as the blank control (CK). Three stress treatments were applied: 4 °C cold stress, 200 mM NaCl-induced salt stress, and 15% PEG-6000-induced osmotic stress (PEG-6000 was used to generate PEG-6000-induced osmotic stress, simulating the osmotic perturbation caused by water deficit). NaCl andPEG-6000 were purchased from Sinopharm Chemical Reagent Co., Ltd., Beijing, China. The stress-treatment conditions used in this study were selected by referring to multiple previously published safflower-related reports from our research group [35,36]. They trigger robust stress-responsive transcription in hydroponic safflower seedlings without causing rapid seedling mortality, and are suitable for time-series leaf sampling for qRT-PCR assays. Functional leaves were harvested at 0, 1, 3, 6, 12, and 24 h after treatment, with an additional 48 h time point specifically set for cold treatment.

For MeJA hormone treatment, the experimental procedure was optimized based on a previously described method [37]. A 100 mM MeJA working solution was prepared, and 0.1% (*v*/*v*) absolute ethanol solution was used as the solvent control. MeJA was obtained from Solarbio Science & Technology Co., Ltd., Beijing, China. Absolute ethanol was purchased from Sinopharm Chemical Reagent Co., Ltd., Beijing, China. Uniform safflower inflorescences at the initial flowering stage were evenly sprayed with corresponding solutions, sealed, and incubated in darkness. Corolla samples were collected at 0, 3, 6, and 12 h post-treatment. All samples were immediately frozen in liquid nitrogen and stored at −80 °C. Three biological replicates were established for each treatment group.

### 4.2. Genome-Wide Identification and Physicochemical Property Analysis of the CtTIFY Gene Family

Based on the published safflower reference genome, *Arabidopsis thaliana* JAZ protein sequences were used as queries for genome-wide homology screening via web-based NCBI BLASTP (BLAST+ 2.16.0, Protein BLAST service, https://blast.ncbi.nlm.nih.gov/Blast.cgi (accessed on 23 March 2026), with an E-value < 1 × 10^−5^. Meanwhile, HMMER 3.0 was employed to search the conserved TIFY (PF06200) and Jas (PF09425) domains. After integrating screening results, domain integrity was further verified using Pfam, SMART, and NCBI CDD databases. Redundant and incomplete sequences were removed to obtain the final Ct*TIFY* family members. The ExPASy-ProtParam online tool was used to analyze basic physicochemical properties of Ct*TIFY* proteins, including amino acid length, molecular weight (MW), theoretical isoelectric point (pI), instability index, aliphatic index, and hydropathicity.

### 4.3. Phylogenetic Tree Construction

JAZ protein sequences of *Arabidopsis thaliana*, *Oryza sativa*, *Helianthus annuus*, and *Carthamus tinctorius* cv. “Yuhonghua No. 1” were retrieved from the following databases: *Arabidopsis thaliana* from TAIR; *Oryza sativa* from Rice Genome Annotation Project and NCBI Assembly database (GCF_001433935.1); *Helianthus annuus* from Plant Genomes Database and NCBI Protein database (KAF5760272.1); *Carthamus tinctorius* cv. “Yuhonghua No. 1” from NCBI SRA database (BioProject: PRJNA642978). Multiple sequence alignment was performed using MUSCLE, and poorly conserved sites were trimmed. A neighbor-joining (NJ) phylogenetic tree was constructed using MEGA 11 with 1000 bootstrap replicates to validate tree topology and phylogenetic reliability.

### 4.4. Chromosomal Localization and Collinearity Analysis of CtTIFY Genes

Chromosomal distribution information of *CtTIFY* genes was extracted from safflower genome annotation files. The chromosomal localization map was visualized using the MG2C_v2.1 online tool (https://qiaoyundeng.github.io (accessed on 8 April 2026)). Intraspecific and interspecific collinearity analyses were performed using MCScanX. The non-synonymous substitution rate (*Ka*), synonymous substitution rate (*Ks*), and *Ka/Ks* ratio of homologous gene pairs were calculated to evaluate the selection pressure driving Ct*TIFY* family evolution (Table S1).

### 4.5. Gene Structure and Conserved Motif Analysis of CtTIFY Genes

Exon–intron structures and untranslated region (UTR) distributions of *CtTIFY* genes were analyzed using GSDS 2.0 (https://gsds.gao-lab.org (accessed on 8 June 2026)). Conserved protein motifs were predicted using MEME 5.5.9 (https://meme-suite.org/meme/ (accessed on 8 June 2026)) with the following parameters: maximum 20 motifs, amino acid length ranging from 6 to 50 aa, and default settings for other parameters. Gene structures and conserved motifs were integrated and visualized using TBtools V2.463 based on phylogenetic clustering results.

### 4.6. Prediction of Promoter Cis-Acting Elements

The 2000 bp upstream sequences of the ATG start codon of each *CtTIFY* gene were extracted and submitted to the PlantCARE database (https://bioinformatics.psb.ugent.be/webtools/plantcare/html/ (accessed on 24 July 2026)) for cis-element prediction. Elements associated with light response, abiotic stress, and phytohormone signaling were screened, statistically analyzed, and visualized as a heatmap using TBtools V2.463.

### 4.7. Transcriptome Expression Profiling Analysis

Two public safflower RNA-seq datasets (PRJNA738310, PRJNA646045) were used to analyze the spatiotemporal expression patterns of *CtTIFY* genes. The datasets covered four flower color phenotypes, high/low linoleic acid seed samples at 10 and 20 days after flowering (DAF), seed germination samples at 1, 3, 5, 7, and 10 days after germination (DAG), and five successive flower developmental stages. Raw reads were quality-controlled and aligned, and FPKM values were calculated for *CtTIFY* genes. Expression data were normalized via log_2_ (FPKM+1), and a clustering heatmap was generated using R software (v4.2.1).

### 4.8. qRT-PCR Validation of Gene Expression

Total RNA was isolated using TRIzol reagent (Pulebo Biotechnology Co., Ltd., Beijing, China), and first-strand cDNA was synthesized via reverse transcription. Reverse transcription was performed using PrimeScript™ RT reagent Kit (Vazyme Biotech Co., Ltd., Nanjing, China). The safflower *Ct60S* gene was used as the internal reference gene, and specific primers for target *CtTIFY* genes were designed (Table S2). qRT-PCR was performed in a 15 μL reaction system with the following program: 95 °C for 3 min, followed by 40 cycles of 95 °C for 10 s and 60 °C for 30 s. qRT-PCR amplification was conducted on a QIAquant 96 2plex Real-Time PCR Detection System (QIAGEN, Hilden, Germany) using SYBR Green Premix Ex Taq™ (GenStar Biosolutions Co., Ltd., Beijing, China). The relative gene expression levels were calculated using the 2^−ΔΔCt^ method, and expression pattern graphs were plotted using GraphPad Prism 8.

### 4.9. Subcellular Localization Assay

The full-length CDS of representative *CtTIFY* genes was amplified and inserted into the 35S::GFP vector to construct 35S::CtTIFY-GFP fusion vectors. Recombinant vectors were transformed into GV3101 (Weidi Biotechnology, Shanghai, China) and transiently infiltrated into *Nicotiana benthamiana* leaves. After 48–72 h of cultivation, GFP fluorescence signals were observed using a laser confocal scanning microscope (Leica, Wetzlar, Germany), with empty 35S::GFP as the negative control.

### 4.10. Statistical Analysis

All experimental data were analyzed using SPSS 27.0. One-way ANOVA followed by the least significant difference (LSD) multiple comparison test was used to evaluate significant differences among groups. A *p* < 0.05 was considered statistically significant. All data are presented as the mean ± SEM of three biological replicates.

## 5. Conclusions

In this study, we performed the first comprehensive genome-wide identification and analysis of the Ct*TIFY* gene family in safflower. A total of 14 *CtTIFY* genes were identified and classified into three subfamilies (JAZ, TIFY, ZML). Evolutionary analysis demonstrated that segmental duplication drives Ct*TIFY* family expansion, and the family has undergone strong purifying selection with significant structural and physicochemical divergence among subfamilies. Multi-omics and qRT-PCR results revealed associations based on transcript expression levels of *CtTIFY* genes with safflower growth, development, quality formation and stress adaptation. *CtTIFY* members exhibit correlations in transcript expression levels with floral development, seed oil accumulation, secondary metabolism and abiotic stress responses. Among them, *CtJAZ1*, *CtJAZ6*, and *CtJAZ8* are key transcriptionally responsive candidate genes, potentially mediating multi-hormone crosstalk and broad-spectrum stress responses. This study characterizes the evolutionary rules and diversity in transcript expression levels of the safflower *CtTIFY* family, enriches Asteraceae TIFY-family research resources, and provides key genetic candidates for future safflower stress-resistance and quality-improvement breeding programs.

## Figures and Tables

**Figure 1:**
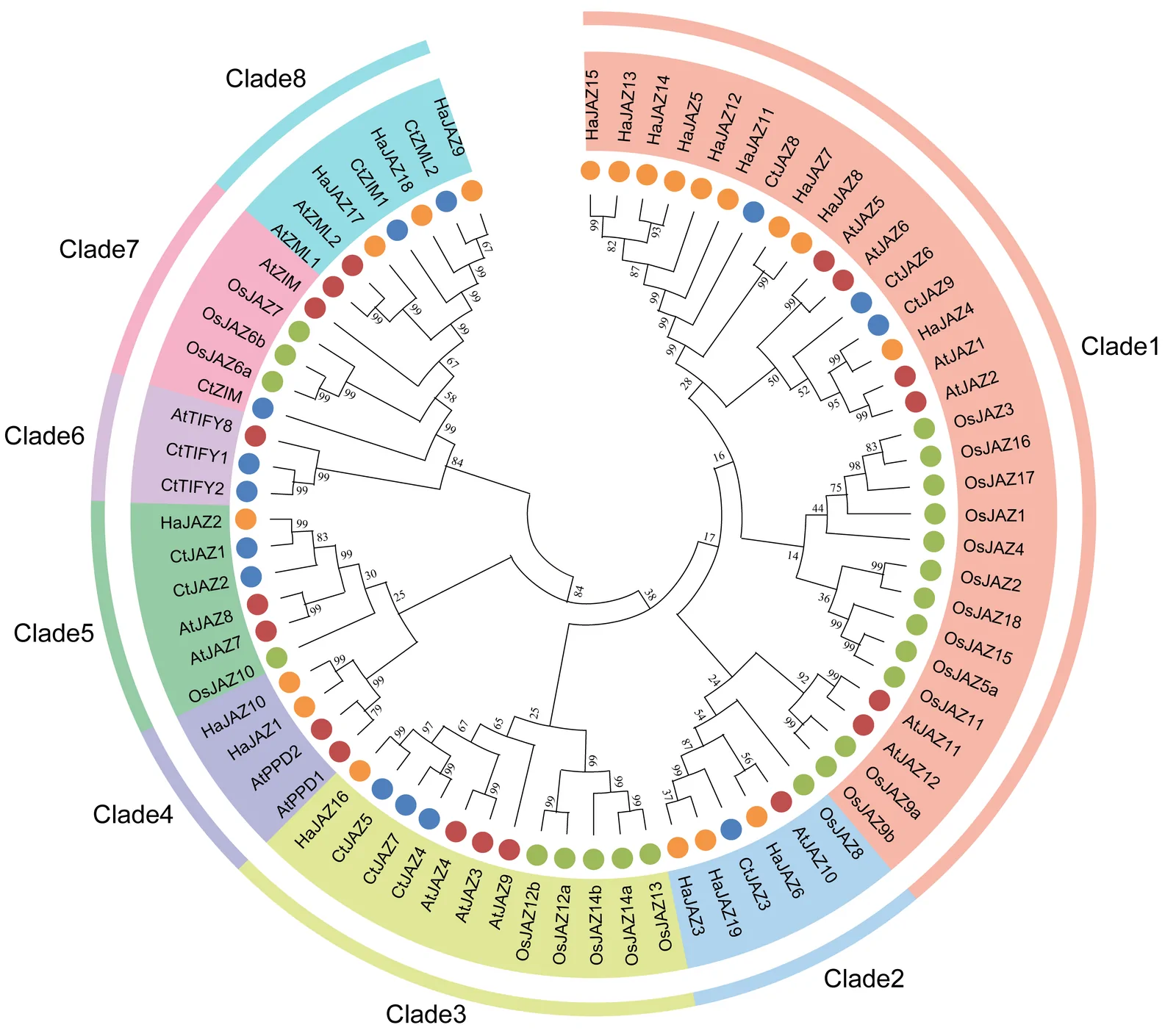
Phylogenetic tree of TIFY proteins from *Carthamus tinctorius*, *Arabidopsis thaliana*, *Oryza sativa*, and *Helianthus annuus*. The neighbor-joining tree was constructed using MEGA 11 with 1000 bootstrap replicates. Different clades are marked with different color backgrounds. Blue dots indicate safflower CtTIFY proteins.

**Figure 2:**
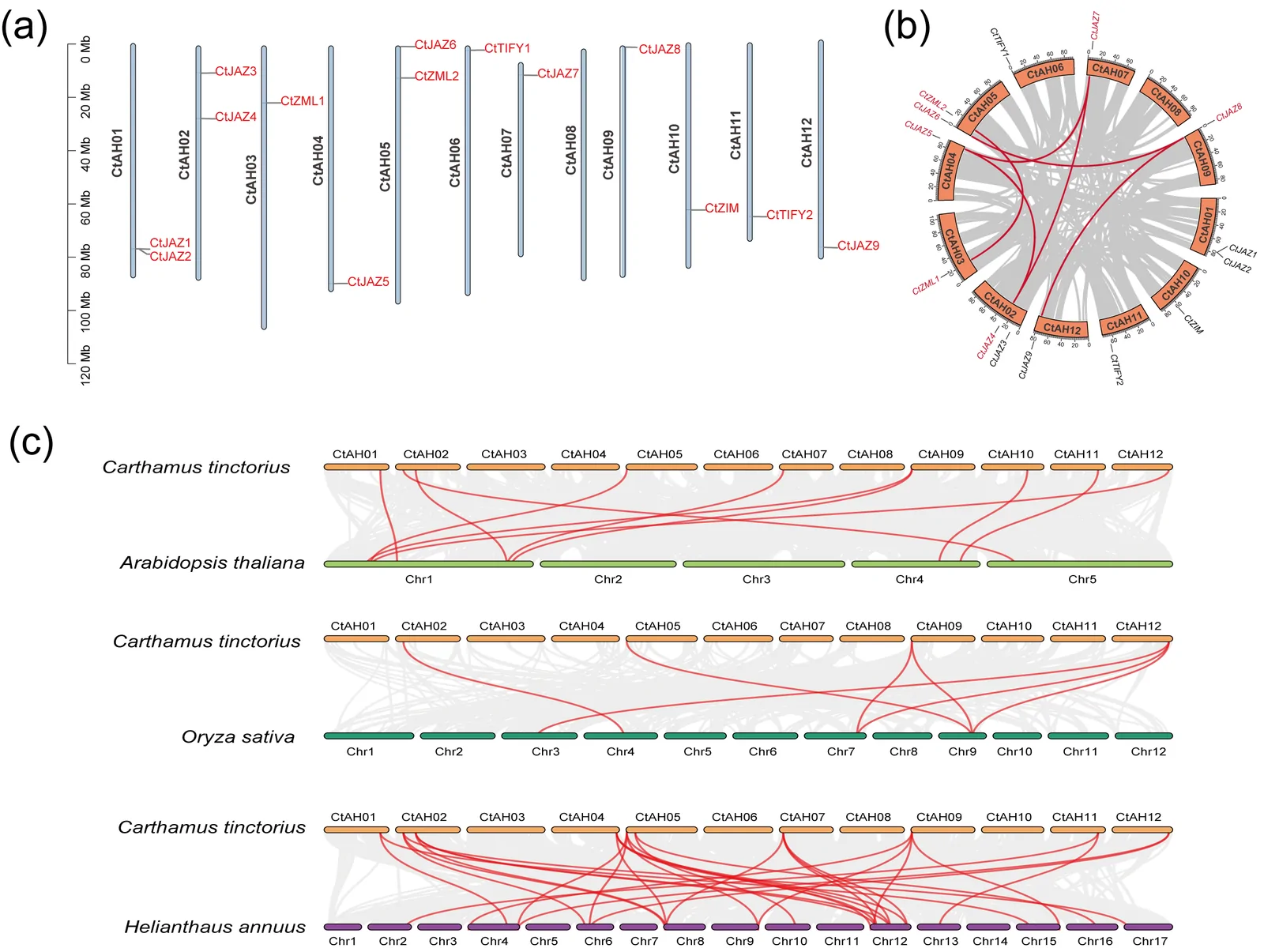
Chromosomal locations and collinearity analysis of the *CtTIFY* gene family. (**a**) *CtTIFY* genes are mapped on chromosomes. (**b**) Collinearity analysis of *CtTIFY* genes. A circle plot was constructed, and the identified collinear genes are linked by red lines. (**c**) Collinearity relationship of *CtTIFY* genes among *Arabidopsis thaliana*, *Helianthus annuus*, and *Oryza sativa*. Identified collinear genes are linked by red lines.

**Figure 3:**
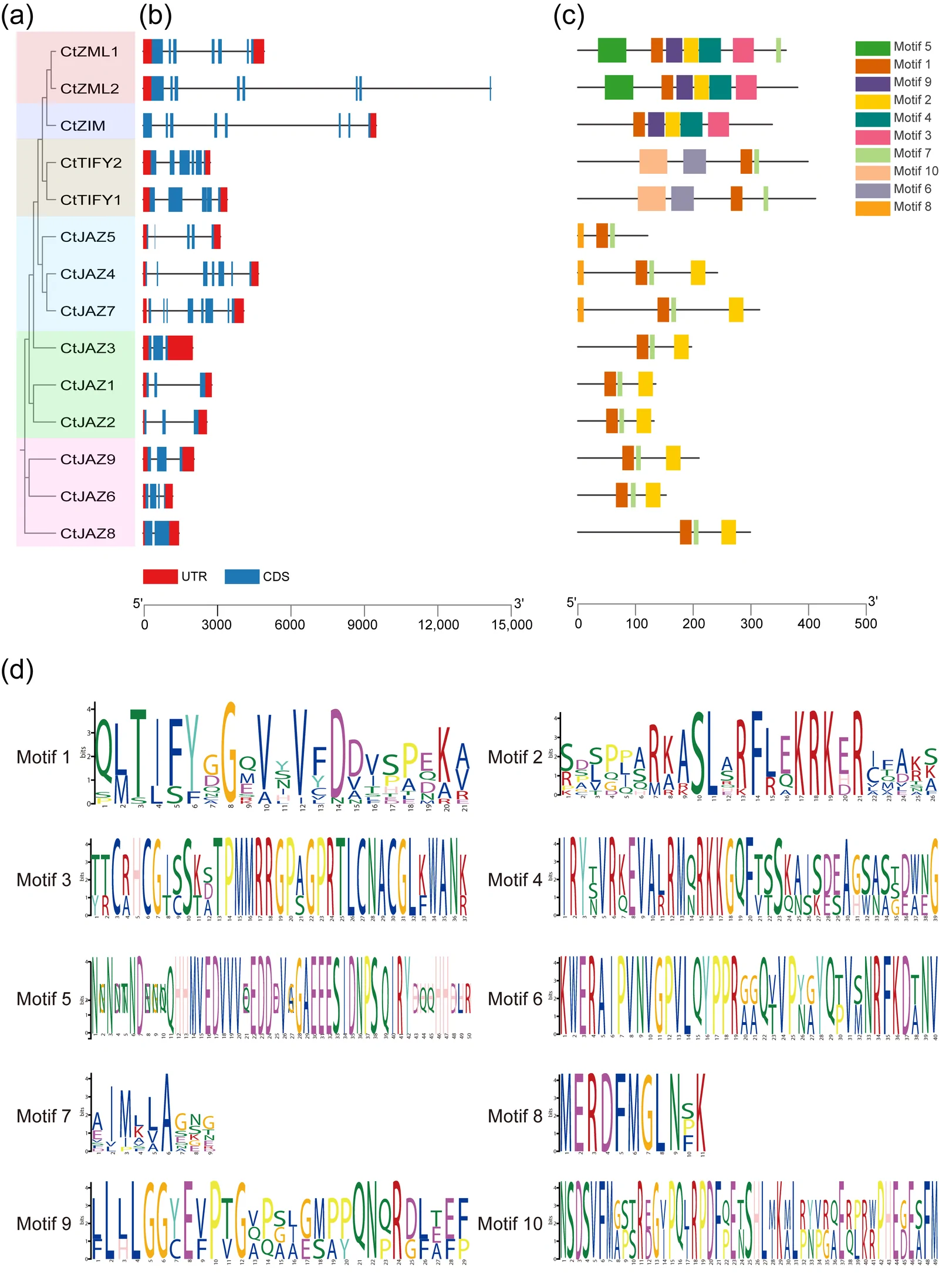
Gene structure, conserved domain and motif analysis of CtTIFY proteins from *Carthamus tinctorius*. (**a**) The phylogenetic tree of all CtTIFY proteins was constructed using the ML method with 1000 bootstrap replicates. (**b**) The UTR and CDS organization of CtTIFYs. The red boxes represent UTRs, and blue boxes represent CDSs. (**c**) The motifs in the CtTIFY proteins were identified by the MEME program. Different motifs numbered 1–10 have different colors, of which sequence logos are shown in (**d**). (**d**) Sequence logos of the ten conserved motifs identified in CtTIFY proteins. Each logo stack indicates an amino acid position, and the height of each letter indicates the relative frequency of the amino acid.

**Figure 4:**
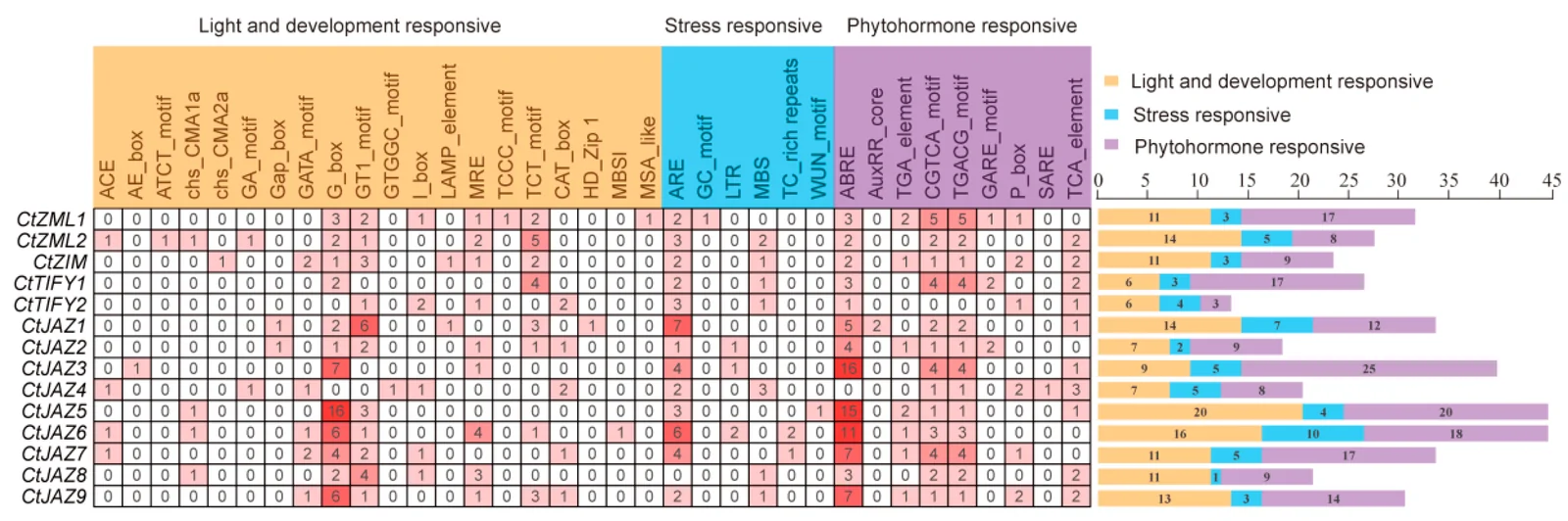
Cis-acting element analysis of *CtTIFY* family gene promoters. Each row represents a *CtTIFY* family gene, and each column represents a specific cis-acting element. Elements are grouped into three categories based on function: light- and development-responsive (yellow background), stress-responsive (blue background), and phytohormone-responsive (purple background). The number in each cell indicates the count of the corresponding element in the gene promoter, with red color intensity positively correlated with the count. The stacked bar chart on the right summarizes the total number of cis-elements of each functional category for every *CtTIFY* gene, adopting the identical three-color classification scheme.

**Figure 5:**
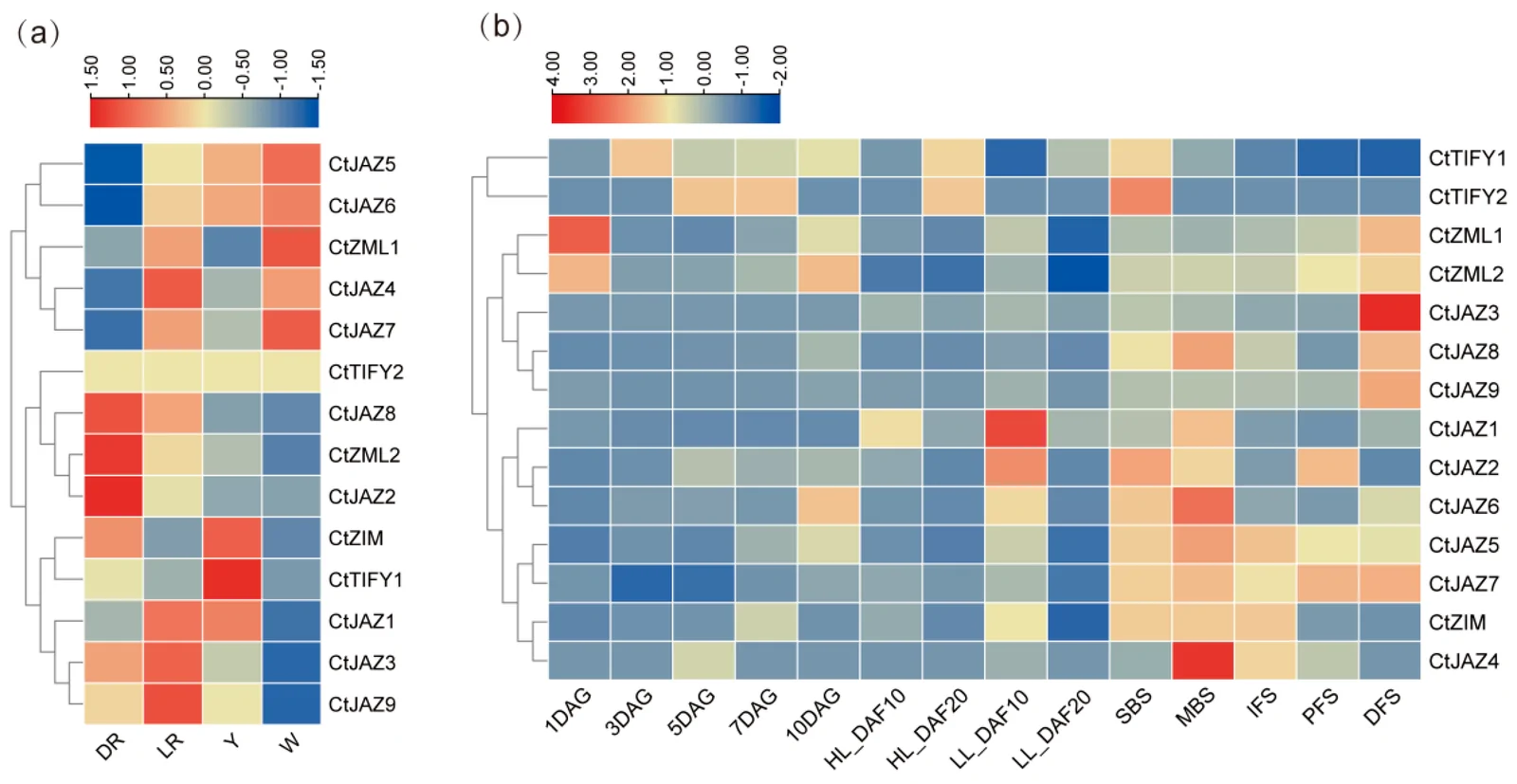
Expression profiles of *CtTIFY* genes in *Carthamus tinctorius*. (**a**) Expression profiles of *CtTIFY* genes in four safflower varieties with distinct flower colors. DR, deep red; LR, light red; Y, yellow; W, white safflower varieties. (**b**) Expression patterns of *CtTIFY* genes across various developmental stages and light treatments. DAG, days after germination; HL, high light; LL, low light; SBS, small bud stage; MBS, middle bud stage; IFS, initial flowering stage; PFS, peak flowering stage; DFS, decayed flowering stage. Relative expression levels were obtained via row-wise normalization of FPKM values and visualized according to the color bar.

**Figure 6:**
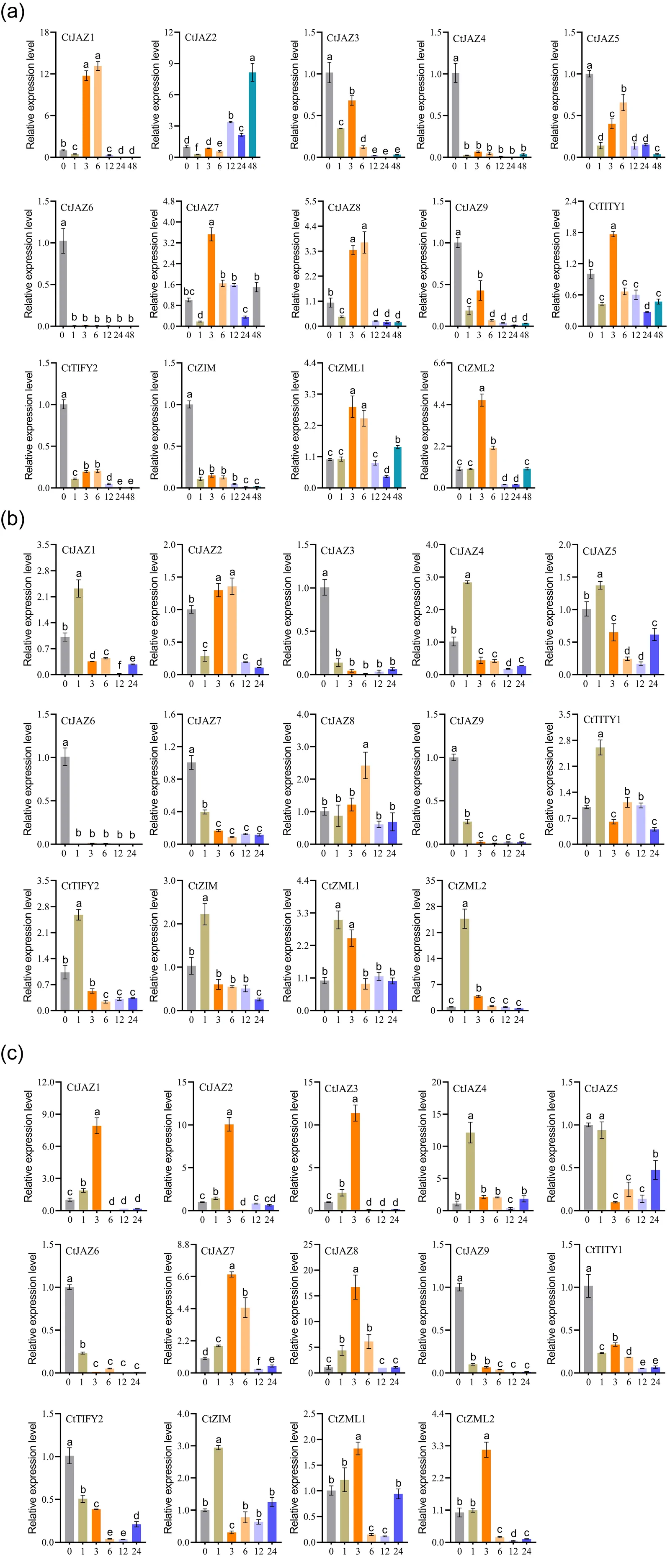
Expression analysis of *CtTIFY* genes following cold, NaCl, PEG-6000 treatments. The cold treatment lasted for 48 h to fully detect gene response. Relative expression levels of *CtTIFY* genes following cold (**a**), NaCl (**b**), and PEG-6000 (**c**) treatments. The *X*-axis represents the RNA samples isolated from leaves of different treatment groups at five time points. From left to right: 0 h (control), 1 h, 3 h, 6 h, 12 h, 24 h. Different lowercase letters indicate significant differences at *p* < 0.05 according to one-way analysis of variance (ANOVA).

**Figure 7:**
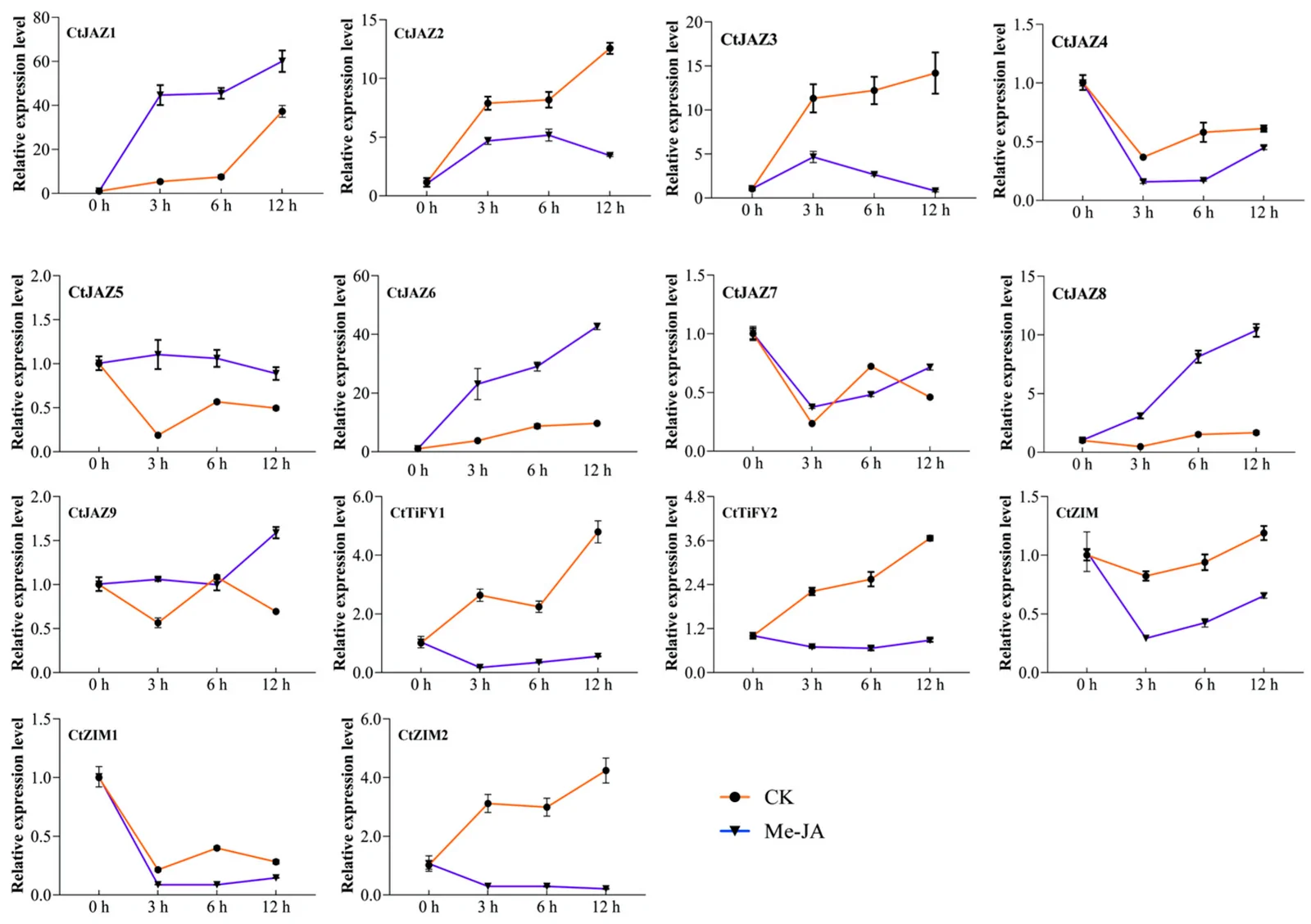
Relative expression profiles of 14 *CtTIFY* genes in safflower corollas at 0, 3, 6 and 12 h after MeJA treatment. Blue lines stand for MeJA group, and orange lines stand for solvent control.

**Figure 8:**
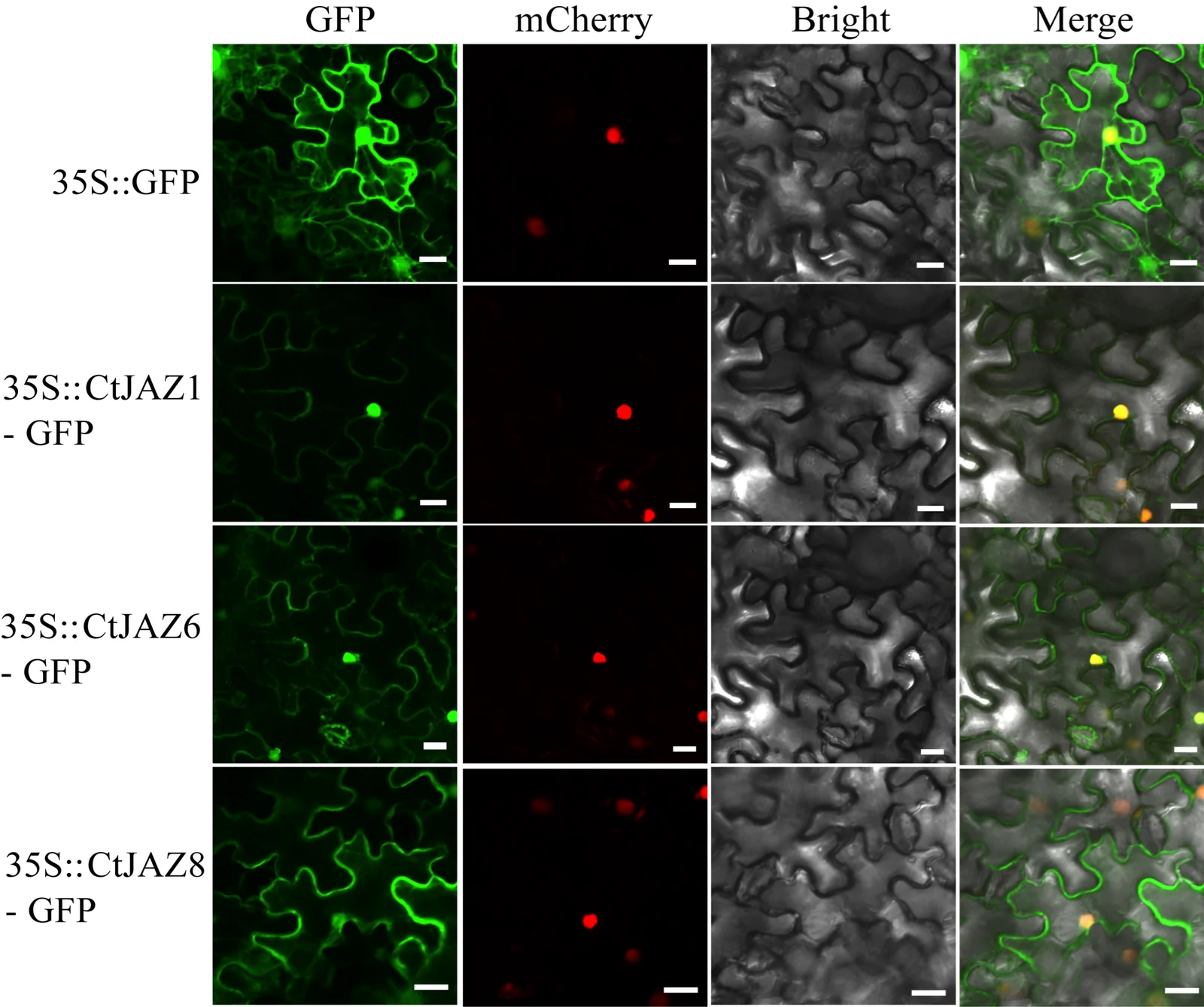
Subcellular localization of *CtJAZ1*, *CtJAZ6* and *CtJAZ8* in *Nicotiana benthamiana* leaf epidermal cells. GFP, green fluorescence signal of fusion proteins; mCherry, red fluorescence signal of nuclear marker; Bright, bright-field image; Merge, merged image of GFP, mCherry and bright field channels. Scale bar = 20 μm.

**Table 1:** Molecular characteristics of *CtTIFY* genes in *Carthamus tinctorius*.

Gene Name	Sequence ID	Number of Amino Acids	Theoretical pI	Instability Index	Aliphatic Index	Grand Average of Hydropathicity	CDS (bp)	MW (kDa)
CtZML1	CtAH03T0096700.1	361	5.73	48.63	64.6	−0.709	1140	39.7
CtZML2	CtAH05T0082800.1	381	5.75	48.68	58.11	−0.912	1200	41.9
CtZIM	CtAH10T0176300.1	337	6.51	27.99	53.26	−0.657	1014	36.9
CtTIFY1	CtAH06T0013400.1	412	9.06	47.96	65.17	−0.428	1086	39.1
CtTIFY2	CtAH11T0211300.1	399	7.87	45.31	58.95	−0.522	1143	41.5
CtJAZ1	CtAH01T0194500.1	135	9.12	98.63	62.07	−0.966	408	15.5
CtJAZ2	CtAH01T0194600.1	132	9.75	66.4	63.64	−0.975	399	15
CtJAZ3	CtAH02T0076300.1	197	9.45	49.72	74.82	−0.294	594	21.1
CtJAZ4	CtAH02T0137500.1	242	9.21	53.41	68.26	−0.277	729	26.1
CtJAZ5	CtAH04T0233400.1	121	5.88	33.64	57.27	−0.631	528	18.6
CtJAZ6	CtAH05T0002300.1	153	5.36	49.11	68.3	−0.682	462	17.1
CtJAZ7	CtAH07T0038000.1	315	8.79	54.12	62.92	−0.28	948	33.3
CtJAZ8	CtAH09T0006500.1	299	8.6	47.85	78.26	−0.449	900	32.5
CtJAZ9	CtAH12T0192200.1	210	8.99	62.24	63.67	−0.655	633	22.9

## Data Availability

Transcriptome sequencing data were obtained from the National Center for Biotechnology Information (NCBI) under the biological project numbers PRJNA646045 (https://www.ncbi.nlm.nih.gov/bioproject/646045 (accessed on 27 July 2026)) and PRJNA738310 (https://www.ncbi.nlm.nih.gov/bioproject/738310 (accessed on 27 July 2026)).

## Supplementary Materials

The following supporting information can be downloaded at https://www.mdpi.com/article/10.3390/ijms27198752/s1.
