# Supplementary material for: Genome-Wide Identification and Functional Characterization of the TIFY Gene Family Associated with Abiotic Stress and Hormone Response in Carthamus tinctorius L
Source: Int J Mol Sci. 2026 Sep 30;27(19):8752. doi: 10.3390/ijms27198752 (PMC13664998; doi:10.3390/ijms27198752)
Supplement: Supplementary file 1 [file ijms-27-08752-s001.zip › ijms-4565883-supplementary.pdf]

Table S1. Ka/Ks ratios of duplicated gene pairs of *CtJAZs*.

Sequence	Method	Ka	Ks	Ka_Ks	P-Value(Fisher)	Purifying Selection
CtZML1-CtZML2	YN	0.10	0.71	0.15	7.6626e-13	YES
CtJAZ4-CtJAZ5	YN	0.34	5.43	0.06	0.00000654	YES
CtJAZ4-CtJAZ7	YN	0.22	0.96	0.22	2.2644e-9	YES
CtJAZ5-CtJAZ7	YN	0.28	1.29	0.21	0.00003904	YES
CtJAZ6-CtJAZ8	YN	0.69	NaN	NaN	0.00169648	YES
CtJAZ8-CtJAZ9	YN	0.67	NaN	NaN	0.00053656	YES

Table S2. The primers used for qRT-PCR in this study.

Gene	Gene ID	Forwaed primer (5′-3′)	Reverse primer (5′-3′)	Length (bp)
CtJAZ1	CtAH01T0194500.1	CAAGAAACACCCAGTGAATT	TGACGTCACAAATGCTAACT	109
CtJAZ2	CtAH01T0194600.1	CAACAGCTAACGATTTTCTATG	CCAGTTGGAGATGGTGAAGA	167
CtJAZ3	CtAH02T0076300.1	GCTTCTCAAGACCGCGATTGC	ACGTCGAAAACCGAGACGCTT	207
CtJAZ4	CtAH02T0137500.1	CAATTTCTGCAACTTCACCAC	CAGTTGTAGACGGAAGATTC	207
CtJAZ5	CtAH04T0233400.1	CAACAATCGTCACTCGACTGA	CTTGGCTAGAAAGGGTCTTG	233
CtJAZ6	CtAH05T0002300.1	AAGCAGATGATCTCCCTTTG	GCCTTGTCGGCTGGAAAATC	178
CtJAZ7	CtAH07T0038000.1	TGCAGTCCACTGGCCTCTGC	ATGGCTCGGTGGTTCCAGCA	278
CtJAZ8	CtAH09T0006500.1	AATGCCAAATTTGATGCTCCA	GTTGTGAGGCCTTTTGGCCTG	118
CtJAZ9	CtAH12T0192200.1	CAACGGCCACCATGAACCTCTT	CTTCATGATCTCCTTCACCTT	158
CtTIFY1	CtAH06T0013400.1	TGGCCAAAGAAACGATTTTTC	CACCATCTCTTGATGAACCCA	104
CtTIFY2	CtAH11T0211300.1	TGAAGGGGTGCCATTCTTTGG	TGGTCGCAACTGGGGGATACC	133
CtZIM	CtAH10T0176300.1	GTGCAAAAGGTGTTTTTACAT	TGGGATCTCATCTTGTGCTGC	78
CtZML1	CtAH03T0096700.1	GAGTTCGGTGAGATTCCGTCG	GCGGCATTATGGCGAGGT	199
CtZML2	CtAH05T0082800.1	GAAGAGTCCATAGACAACCCT	GTCCGGTACTGCATACAAAGC	156
Ct60s		CATCCATTATCCAACAATC	AAGAGTAATCAGTCTCCA	

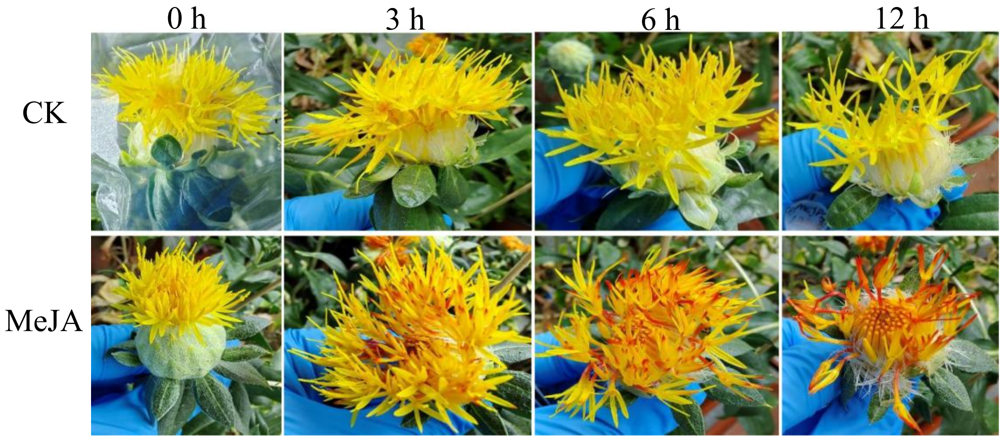

Figure S1. Phenotypic changes of safflower corollas under MeJA and 0.1% ethanol control at 0, 3, 6 and 12 h.
